# Genomic regions associated with tuber traits in tetraploid potatoes and identification of superior clones for breeding purposes

**DOI:** 10.3389/fpls.2022.952263

**Published:** 2022-07-22

**Authors:** Jeewan Pandey, Douglas C. Scheuring, Jeffrey W. Koym, M. Isabel Vales

**Affiliations:** ^1^Department of Horticultural Sciences, Texas A&M University, College Station, TX, United States; ^2^Texas A&M University AgriLife Research and Extension Center, Lubbock, TX, United States

**Keywords:** tuber morphology, genome-wide association studies, single-nucleotide polymorphism, *Solanum tuberosum* ssp. *tuberosum* L., genomic prediction

## Abstract

In potato breeding, morphological tuber traits are important selection targets to meet the demands of the fresh and processing markets. Understanding the genetic basis of tuber traits should guide selection and improve breeding efficiencies. However, this is challenging in potato due to the complexity of the traits and the polyploid nature of the potato genome. High-throughput affordable molecular markers and new software specific for polyploid species have the potential to unlock previously unattainable levels of understanding of the genetic basis of tuber traits in tetraploid potato. In this study, we genotyped a diversity panel of 214 advanced clones with the 22 K SNP potato array and phenotyped it in three field environments in Texas. We conducted a genome-wide association study using the GWASpoly software package to identify genomic regions associated with tuber morphological traits. Some of the QTLs discovered confirmed prior studies, whereas others were discovered for the first time. The main QTL for tuber shape was detected on chromosome 10 and explained 5.8% of the phenotypic variance. GWAS analysis of eye depth detected a significant QTL on chromosome 10 and explained 3.9% of the phenotypic variance. Our study found that multiple QTLs govern russeting in potato. A major QTL for flesh color on chromosome 3 that explained 26% of the phenotypic variance likely represents the *Y* locus responsible for yellow flesh in potato tubers. Several QTLs were detected for purple skin color on chromosome 11. Furthermore, genomic estimated breeding values were obtained, which will aid in the early identification of superior parental clones that should increase the chances of producing progenies with higher frequencies of the desired tuber traits. These findings will contribute to a better understanding of the genetic basis of morphological traits in potato, as well as to identifying parents with the best breeding values to improve selection efficiency in our potato breeding program.

## Introduction

Domestication and selective breeding have resulted in significant morphological variation in organ shape in cultivated plant species (Sun et al., 2017). Examples of studies on phenotypic diversity and their genetic basis include grain size and shape in wheat (Gegas et al., 2010), fruit shape in tomato and melon (Monforte et al., 2014), and phenotypic diversification in *Brassica rapa* and *Brassica oleracea* (Cheng et al., 2016). Potato domestication took place around 8,000 years ago from a wild diploid *Solanum* species in an area located on the border of present-day Peru and Bolivia (Spooner et al., 2005). Modern cultivars are the result of extensive crossbreeding between cultivar groups as well as wild species. Andean tetraploid varieties likely resulted from repeated sexual polyploidization of early landrace diploids (Spooner et al., 2014). The domestication of potatoes involved selection of shorter stolons, larger tubers, diverse tuber shapes, and reduction of bitter tuber glycoalkaloids (Spooner et al., 2005).

Potato breeding activities are mainly concentrated on developing cultivars for the processing and table/fresh markets. Processing potatoes are used to produce chip, French fries, and dehydrated products. Table/fresh market types are further grouped as reds, russets, whites, purples, and yellows (based on skin or flesh color). Market-group-specific morphological traits include tuber shape, eye depth, degree of russeting, tuber number, tuber weight, skin color, and flesh color. Tuber shape is extremely important and is based on historical regional preferences coupled with local culinary practices (Stark et al., 2020). Chips are made with round tubers, whereas French fries are made with oblong to long tubers. Likewise, consumers expect red and yellow table stock cultivars to be round or oval (Chen et al., 2018). The irregular shape and deep eyes in potatoes lead to higher costs due to significant peeling losses and thus are undesirable traits (Li et al., 2005; ŚLiwka et al., 2008). Another trait, skin russeting texture is an inherited characteristic in some potato cultivars such as Russet Burbank and Russet Norkotah (De Jong, 1981). Russet skin is not present in native potatoes. In Europe, smooth skin texture has been preferred by consumers, whereas russet skin is desired in the USA and Canada. Tuber size, skin color, and flesh color traits are also preferred by consumers and industry depending on the use of potatoes. Thus, breeding for tuber morphological traits in potatoes is crucial as these traits affect consumption.

The cultivated potato is a vegetatively propagated crop with a complex autotetraploid (2*n* = 4x = 48) genome, a basic chromosome number of 12, and a genome size of 844 Mb (Xu et al., 2011). Due to high heterozygosity, broad segregation for many traits will occur when breeders make crosses (Bonierbale et al., 2020). Some of these traits are poorly characterized and their heritability is often low (Thiele et al., 2021). Due to this complexity in autopolyploids, most molecular analytical tools and software have been limited to diploids (Bourke et al., 2018a). Utilizing bi-parental populations in diploid potatoes, quantitative trait loci (QTL) mapping studies have been performed to map several tuber quality traits. Tuber shape inheritance, whether it is monogenic or polygenic, has been inconsistent. van Eck et al. (1994a), using restriction fragment length polymorphisms (RFLPs), identified a single locus, *Ro*, that explains the inheritance of qualitative tuber shape, with round being dominant over long on chromosome 10. Other reports using populations with different genetic backgrounds mention QTLs on chromosomes 2, 5, and 11 (Bradshaw et al., 2008), and 2 and 11 (ŚLiwka et al., 2008). Eye depth was linked to the *Ro* locus on chromosome 10 (Li et al., 2005; ŚLiwka et al., 2008). Similarly, using QTL analysis, a dominant allele at the *Y* (yellow) locus on potato chromosome 3 was identified for the yellow flesh color (Bonierbale et al., 1988). A beta-carotene hydroxylase gene (*BCH*) was found in the same position as the *Y* locus, suggesting that this is the most likely candidate gene for yellow flesh (Thorup et al., 2000; Kloosterman et al., 2010). Anthocyanin pigments accumulate in the tuber skin and flesh, giving it a red or purple color (Eichhorn and Winterhalter, 2005). Anthocyanin pigmentation in tuber skin was first mapped on chromosome 10 by van Eck et al. (1994b). The *Pf* locus is tightly linked to the *I* locus (encoding an MYB transcription factor) and regulates the synthesis of purple and red anthocyanins (De Jong, 1987; De Jong et al., 2004). The *R* locus, involved in the biosynthesis of red anthocyanins was first mapped on chromosome 2 by van Eck et al. (1993) and it encodes dihydroflavonol 4-reductase (*dfr*) (De Jong et al., 2003; Zhang et al., 2009). The *P* locus involved in the production of blue/purple anthocyanins in potato was first mapped by van Eck et al. (1993) on chromosome 11. Jung et al. (2005) reported that the *P* locus encodes flavonoid 3^′^,5^′^-hydroxylase (*f3^′^5^′^h*) gene and it is expressed in the tuber skin only in the presence of *I* locus.

High-throughput and affordable molecular markers and recent analytical platforms suitable for the analysis of polyploid species hold the promise of unlocking previously unattainable levels of understanding of the cultivated tetraploid potato genome. TetraploidSNPMap (Hackett et al., 2017) uses allelic dosage to carry out linkage analysis and QTL mapping in autotetraploid species. Other polyploid mapping softwares include the PERGOLA package in R (Grandke et al., 2017), polymapR (Bourke et al., 2018b), and MAPpoly (Mollinari and Garcia, 2019). Advancements in tools for genomic analysis in polyploid species have aided in the identification of QTLs that account for significant amounts of phenotypic variance within a polyploid population. However, underestimation the QTL number, effect, and phenotypic variance explained, and low resolution of individual QTL in a bi-parental population often obstruct causal gene identification (Zhu et al., 2008).

Another powerful strategy to discover markers linked to complex traits is to perform Genome-Wide Association Studies (GWAS) (Cortes et al., 2021). GWAS is an effective method for circumventing many of the limitations of bi-parental linkage mapping. It takes advantage of genetic variation that exists among natural or developed populations utilizing historical recombination and knowledge of population structure (Ortiz, 2020). GWAS pinpoints QTLs by analyzing marker-trait associations that can be attributed to the strength of linkage disequilibrium (LD) between markers and functional polymorphisms across a set of diverse germplasm (Zhu et al., 2008). One challenge in applying GWAS to polyploid species is how to define relatedness between polyploid individuals (i.e., how to generate the kinship matrix, K). GWAS can now account for the kinship matrix (K) in potatoes using GWASpoly (Rosyara et al., 2016). GWASpoly also considers allelic dosage (AAAA, AAAa, AAaa, Aaaa, aaaa). For computational efficiency, Yang et al. (2014) proposed the leave-one-chromosome-out (LOCO) method. *In LOCO* method, a different covariance matrix is calculated for each chromosome based on the markers from all other chromosomes (Yang et al., 2014) and is now implemented in version 2 of GWASPoly. Sharma et al. (2018) examined various GWAS models in cultivated potato genotypes using the Infinium 8 K Potato SNP Array and found that kinship, not population structure, was the most important factor in determining the extent of false associations. Klaassen et al. (2019) evaluated a panel of 277 varieties using SolSTW 20 K Infinium SNP marker array (Vos et al., 2015) and revealed four QTLs for protein content in tetraploid potato. Similarly, Zia et al. (2020) performed a GWAS using SolCAP 12 K array for various morpho-agronomic traits in a panel of 237 tetraploid potato genotypes. Kaiser et al. (2020) used GWAS to identify genetic features associated with common scab resistance in the tetraploid population using the Illumina Infinium 8,303 Potato Array. Recently, Yousaf et al. (2021) reported SNPs associated with potato stolon traits and root traits.

For practical breeding applications, genomic selection (GS) based on genome-wide marker effects is a very promising marker-assisted selection procedure that should speed up the selection of the best candidate clones (to advance in the breeding program or to be used as parents) and thus contributes to increasing genetic gains. Genomic selection is based on genomic estimated breeding values (GEBV) that are predicted from high-density DNA markers dispersed across the genome (Meuwissen et al., 2001). Genomic selection has been effectively applied in animal breeding (Meuwissen et al., 2016) as well as in plant breeding, particularly in cereals like wheat (Poland et al., 2012; Bassi et al., 2016), maize (Bernardo and Yu, 2007; Crossa et al., 2017) and barley (Lorenz et al., 2012). Genomic selection is still in its infancy for many crops and its promise for cultivar improvement has been demonstrated by recent studies in potato (Habyarimana et al., 2017; Sverrisdóttir et al., 2017; Endelman et al., 2018; Bradshaw, 2021; Selga et al., 2021; Wilson et al., 2021). Genetic variance partitioning and genome-wide prediction can be done with allele dosage information (Endelman et al., 2018). GEBV will allow selection of superior parents or advancement of better clones for next-generation to be faster and more effective than using phenotypic information alone.

The Texas A&M University Potato Breeding Program has a collection of 214 advanced clones selected over 40 years and maintained *in vitro* (Pandey et al., 2021). The clones have been propagated and evaluated under field conditions. Since there is variation in tuber morphology traits, this panel can be used to explore genomic regions associated with tuber traits and to identify superior individuals to use as parents. Recent molecular marker-dense platforms and analytical software optimized to conduct statistical genomic analysis in polyploid species are expected to provide good allelic calls and better calculations of relatedness, handle additive and dominant models, as well as allow more accurate detection of QTL number, position, effects, and phenotypic variance explained. In this study, we aimed to detect QTLs associated with tuber shape, eye depth, degree of russeting, tuber number, tuber weight, skin color and flesh color, and to identify clones with superior tuber traits to be use in the breeding program based on genomic estimated breeding values.

## Materials and methods

### Plant materials

The association panel consisted of 214 tetraploid potato clones including advanced clones entered into tissue culture over several decades, varieties released by the Texas A&M Potato Breeding Program, and reference varieties for various market groups. The collection comprised 31 chipping, 62 russet, 32 yellow-skinned, 68 red-skinned, and 21 purple-skinned clones (Pandey et al., 2021). The reference varieties evaluated were: Russet Norkotah (standard, fresh market russet), Atlantic (chipper), Russet Burbank (processing russet), White LaSoda (a white-skinned mutant of Red LaSoda selected by the TAMU Program, fresh market white flesh), and a Yukon Gold strain (TXYG79, fresh market yellow flesh). Analysis of population structure and discriminant analysis of principal components in the panel displayed three sub-populations, as reported earlier (Pandey et al., 2021).

### Genotyping

Genomic DNA was extracted from 50 to 80 mg of fresh young potato leaves from tissue culture plantlets using the DNeasy Plant Pro Kit (Qiagen, Valencia, CA, United States). Samples were assayed using the Infinium 22 K V3 Potato Array on the Illumina iScan (Illumina Inc., San Diego, CA, United States) at Michigan State University. The marker dataset was filtered for polymorphism and minor allele frequency as described in Pandey et al. (2021).

### Field experiments and phenotyping

Three field experiments were conducted in Texas to obtain tuber traits data. In 2019, tuber traits were evaluated in Dalhart (35°58′N, 102°44′W; DAL 2019), and in 2020, the traits were evaluated in Springlake (34°6′N, 102°19′W; SPR 2020) and Dalhart (DAL 2020). In all three environments, entries were planted in 12-hill plots with two replications in a randomized complete block design. Seed tuber pieces were planted with 30 cm spacing between hills and 70 cm spacing between rows. In Springlake, trials were planted in late March and harvested in early July, whereas in Dalhart, trials were planted in early May and harvested in early September, with vine desiccation 2–3 weeks before harvest. Standard potato production practices were followed during the growing period in all years (details are in the 2019 and 2020 reports of Texas A&M Potato Breeding Program: https://potato.tamu.edu/reports/). The environmental conditions during the potato growing season were representative of the subregion (High Plains) which is characterized by a cold semi-arid climate. Temperature and precipitation regimes during the growing season can be obtained from the 2019 and 2020 reports of the Texas A&M Potato Breeding Program: https://potato.tamu.edu/reports/. Rainfall was supplemented with center pivot irrigation.

For the traits assessed visually, the scores were given by replication and were representative of all tubers harvested per plot. Tuber shape was determined using a 1 to 5 scale, where 1 = round, to 5 = long. Tuber shape was also measured as the ratio of tuber length to width (roundness score) and tuber length to thickness (flatness score). Length (mm) of the tuber was the distance from the stolon end to the bud end of the tuber, width (mm) was the highest dimension in the equatorial area of the tuber, and thickness (mm) was measured perpendicularly to width. For each clone, length, and width data were collected from five tubers (from the 113.4 to 170.1 g grading group) per replication using a bar-coded ruler. Eye depth was evaluated on a 1–5 scale, where 1 = very deep eyes, to 5 = very shallow eyes. The degree of skin russeting texture was evaluated on a 1–5 scale, where 1 = no russeting (smooth) to 5 = heavy russetting. Grading score was obtained on a 1–5 scale, where 1 = poor, to 5 = excellent. Average weight per tuber was calculated by dividing the total tuber yield of the plot by the number of tubers; average tuber number per plant was calculated as a total number of tubers harvested per plot per replication divided by plant stand at 60 DAP (days after planting), and the average tuber weight per plant was calculated from the total tuber yield of the plot divided by the plant number plant stand at 60 DAP.

Objective color measurements for the skin and flesh color were obtained from three tubers per plot per replication with a colorimeter, Konica–Minolta Chroma Meter CR-400 with 8-mm aperture and 0° viewing angle (Konica-Minolta, Inc., Tokyo, Japan), using the International Commission on Illumination (CIE) 1976 L^*^a^*^b^*^ color spaces. The instrument was calibrated against a standard white reference tile provided by the instrument manufacturer. CIE 1976 L^*^a^*^b^*^ is a three-dimensional color space where L^*^ (lightness) represents the white to black axis, a^*^ represents the red to green axis, and b^*^ represents the yellow to blue axis. The colorimeter collected an average of three readings per tuber, and each reading covered 50.3 mm^2^ (8 mm diameter area) of the surface. Data were recorded using Color Data Software CM-S100w SpectraMagic NX (Version 2.8). The color was expressed in LCH color space, in which L^*^ is lightness, C is chroma (saturation) and H is hue angle. Purple skin in tuber was scored as a binary trait (Purple/Non-purple).

### Statistical analyses

Analysis of variance across environments (DAL 2019, DAL 2020, and SPR 2020 - combined) and by individual environment, was done for each tuber trait using mixed models in JMP Pro 16® (SAS Institute, Cary, NC). Clones were treated as fixed effects, while the environment, replication within environments, and their interaction were treated as random effects. To determine the significance of location (Dalhart *vs* Springlake) and location by clone interactions, the DAL 2020 and SPR 2020 datasets were used; locations were treated as fixed effects. To ascertain the significance of year, and year by clone interaction, analysis of variance was done for 2 years at the Dalhart location using the DAL 2020 and DAL 2020 datasets; years were considered random effects.

Tuber morphological traits were also analyzed separately for each of the three environments (location-year) and combined across environments using the software package META-R (Alvarado et al., 2020). Best linear unbiased estimates (BLUEs), range, LSD, and CV were generated using standard procedures implemented in META-R. Phenotypic correlations between pairs of traits were calculated as simple Pearson correlations. Broad-sense heritability of a given trait in an individual environment (repeatability) was calculated on a genotype mean basis as:


$$H=\frac{\sigma_{2}^{G}}{\sigma_{2}^{G}+\frac{\sigma_{2}^{e}}{r}}X\;100$$

where $\sigma_{2}^{G}$ is the genotypic variance, $\sigma_{2}^{e}$ is the error variance, and r is the number of replications.

Broad-sense heritability on a genotype mean basis for the combination of all environments was calculated as:


$$H=\frac{\sigma_{G}^{2}}{\sigma_{G}^{2}+\left(\sigma_{\mathrm{GE}}^{2}/n\right)+\left(\sigma_{e}^{2}/rn\right)\;}\;X\;100$$

where $\sigma_{G}^{2}$ is the genotypic variance, $\sigma_{\mathrm{GE}}^{2}\;$ is the variance of the interaction of genotype and environment, $\sigma_{e}^{2}$ is the variance of the experimental error, r is the number of replications, and n is the number of environments.

BLUEs were calculated for each genotype, considering the effects of genotypes as fixed. BLUEs used for GWAS for a single environment were estimated as:


$$\;Y_{\mathrm{ik}}=\mu +R_{i}+G_{k}+\varepsilon_{ik}$$

where *Y_ik_* is the value for the trait of interest, *μ* is the mean trait effect, *R_i_* is the effect of the *i*^th^ replicate, G_k_ is the effect of the *k^th^* genotype, and *ε_ik_* is the error associated with the *i*^th^ replication, and the *k^th^* genotype.

Likewise, BLUEs used for GWAS for the combined environment considering genotypes fixed was estimated as:


$$Y_{ijr}=m+G_{i}+E_{j}+R_{r}\left(E\right)+GE_{ij}+e_{ijr}$$

where *Y_ijr_* = trait value for the genotype i in the environment j and rep r, *m* = grand mean of trait, *G* = genotype, *E* = environment, *R(E)* = replication within an environment, e = random error.

Narrow-sense heritability was calculated using a two-stage approach described by Damesa et al. (2017), and ASReml-R (VSN International, UK) for variance component estimation.

### Assessment of linkage disequilibrium and genome-wide association studies

Linkage disequilibrium was estimated for all SNPs with pairwise correlation coefficient (*r*^2^) based on allele frequencies (Hill and Robertson, 1968) using LD.plot function in GWASpoly (Rosyara et al., 2016). A monotone decreasing, convex spline was fit using the R package scam. The appropriate window size to filter the most significant markers was determined based on the extent of LD in the panel, visualized using the function LD.plot.

The whole marker dataset was filtered for polymorphisms and minor allele frequencies and 10,116 SNPs were retained for further analysis. Association analysis was performed with the BLUEs for tuber traits with 10,116 SNPs using the GWASpoly Version 2 package in R (Rosyara et al., 2016). To control population structure, the leave-one-chromosome-out (LOCO) method (Yang et al., 2014) was used. Additive and dominant genetic models were tested for each trait. A Bonferroni test was run for each trait and year to establish a LOD threshold corresponding to a genome-wide false-positive rate of 5%. Manhattan plots were produced using GWASpoly. The proportion of phenotypic variance explained by significant SNPs was estimated using the function fit. QTL in GWASpoly based on the change in the likelihood obtained from backward elimination (Sun et al., 2010). Contextual sequences of candidate SNPs were used in a BLAST search of DM1–3 pseudomolecules (Version 4.03) in the Spud DB database (http://solanaceae.plantbiology.msu.edu/) to identify putative candidate genes.

### Genomic estimated breeding values

Genetic variance partitioning and genome-wide prediction was done with allele dosage information (Endelman et al., 2018).^1^ The BLUEs were used as the response variable for partitioning of variance. The model was


$$\mathrm{BLUE}\left[g_{ij}\right]=\mu_{j}+\underset{k}{\sum }\beta_{k}w_{ik}+a_{i}+d_{i}+s_{ij}+\varepsilon_{ij}$$

where $g_{ij}$ is the genotypic value for clone *i* in year *j*, $\mu_{j}$ is the fixed effect for year *j*, $w_{ik}$ is the (centered) allele dosage for clone *i* at marker *k*, and $\beta_{k}$ is the fixed effect for marker *k*. The vector of additive values, **a**, follows a multivariate normal distribution with covariance proportional to a genomic relationship matrix (**G**) estimated from markers (VanRaden, 2008; Endelman et al., 2018). Non-additive effects were captured using independent and identically distributed residual genetic values, $d_{i}$ . The s_ij_ random has no free variance parameters and follows a multivariate normal distribution (Damesa et al., 2017). The model residuals $\varepsilon_{\mathrm{ij}}$ represent the genotype x year interaction.

Well-established methods were utilized to compute best linear unbiased predictors (BLUP) and standard errors. For a generic random vector **u**, BLUP $\left[u\right]=\hat{u}=CPy$ , where C=cov[u,y] , $P=V^{-1}-V^{-1}X{\left(X^{\prime }V^{-1}X\right)}^{-1}X^{\prime }V^{-1}$ , **V** is the variance–covariance matrix of the response variable, and **X** is the incidence matrix for fixed effects (Searle et al., 1992). The squared correlation with the true value, which equals $r_{i}^{2}=\frac{\mathrm{var}\left[{\hat{u}}_{i}\right]}{\mathrm{var}\left[u_{i}\right]},$ and $\mathrm{var}\left[\hat{u}\right]=CPC^{\prime }$ , represents the reliability of $\hat{u}i$ .

## Results

### Phenotypic data analysis

**Figure 1:**
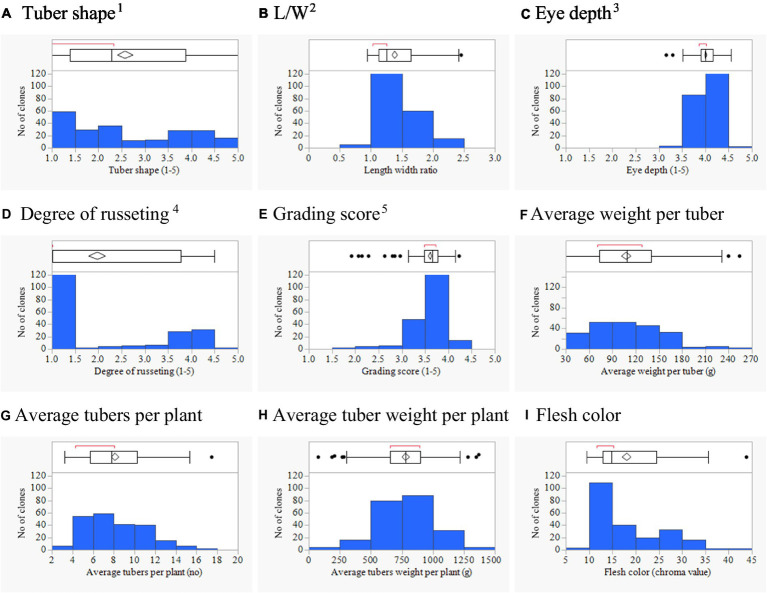
Phenotypic distributions of 214 potato clones for tuber shape **(A)**, L/W **(B)**, eye depth **(C)**, degree of russeting **(D)**, grading score **(E)**, average weight per tuber **(F)**, average tuber number per plant **(G)**, average tuber weight per plant **(H)**, and flesh color **(I)** combined across three environments in Texas: Dalhart 2019, 2020 and Springlake 2020. ^1^1 = round to 5 = long; ^2^1 = deep to 5 = shallow; ^3^1 = none to 5 = heavy; ^4^1 = poor, 5 = excellent; and L/W = length–width ratio.

The potato association mapping panel showed variation for tuber morphology traits and represents a promising resource for GWAS (Table 1). The frequency distribution of phenotypic data showed that most tuber traits were not normally distributed (Figure 1). For the eye depth, the distribution was skewed toward superficial (shallow) eyes. In the case of the degree of russeting, most clones had either smooth or russet skin, with few clones with intermediate or buff type skin. Large variation was found for average tuber weight from very small to large. A larger tuber is often preferred, but there is a growing market for small size potatoes; in the latter case, a high tuber number is desired.

**Table 1:** Summary statistics based on best linear unbiased estimators (BLUEs), broad-sense and narrow-sense heritability for tuber morphological traits of 214 potato clones evaluated in three environments (Env.) in Texas: Dalhart 2019 (DAL 2019), Dalhart 2020 (DAL 2020), and Springlake 2020 (SPR 2020).

	Env.	Tuber shape^1^	L/W	Eye depth^2^	Degree of russeting^3^	Grading score^4^	Av. weight per tuber	Av. tubers per plant	Av. tuber weight per plant	Flesh color
		(1–5)	(ratio)	(1–5)	(1–5)	(1–5)	(g)	(no)	(g)	(chroma value)
BLUEs	DAL 2019	2.7	1.4	4.0	1.9	3.5	111.8	8	804.2	19.6
	DAL 2020	2.7	1.4	4.0	2.1	3.7	124.9	8	931.8	17.7
	SPR 2020	2.4	1.4	4.0	1.9	3.6	87.8	9	650.8	17.1
	Overall	2.6	1.4	4.0	2.0	3.6	107.4	8	788.9	18.1
Range	DAL 2019	1–5	0.8–2.7	3.2–4.8	1–4.5	1.0–4.6	8–298	2–19	67–1907	11–52
(min-max)	DAL 2020	1–5	0.8–2.8	3.0–4.6	1–4.6	2.5–4.6	11–270	3–19	178–1568	8–46
	SPR 2020	1–5	0.9–2.8	3.5–5.0	1–4.6	2.0–4.5	8–232	3–19	77–1264	7–36
	Overall	1–5	0.8–2.8	3.0–5.0	1–4.6	1.0–4.6	8–298	2–19	67–1907	7–52
LSD	DAL 2019	0.5	0.2	0.2	0.3	0.5	46.5	5.9	537.9	4.4
	DAL 2020	0.5	0.2	0.1	0.3	0.4	39.7	3.9	453.6	3.1
	SPR 2020	0.5	0.3	0.1	0.2	0.4	27.3	4.6	309.3	3.7
	Overall	0.6	0.2	0.2	0.3	0.4	32.9	3.4	354.2	2.4
CV	DAL 2019	9.0	8.3	2.8	6.5	7.6	20.3	35.0	33.3	10.9
	DAL 2020	9.3	8.8	1.8	6.2	4.9	15.9	24.1	24.6	8.7
	SPR 2020	11.3	12.1	1.3	5.1	5.4	15.7	27.0	23.9	11.0
	Overall	9.8	9.4	2.0	5.9	6.0	17.7	28.9	27.9	10.3
*H* (%)	DAL 2019	98.4	94.8	90.0	99.5	82.4	89.4	70.8	69.0	95.7
	DAL 2020	98.1	93.7	92.8	99.6	82.7	91.3	79.1	63.1	97.6
	SPR 2020	97.9	89.2	97.8	99.7	82.5	93.8	74.6	72.2	95.9
	Overall	97.3	97.6	68.0	99.1	64.7	91.8	80.3	64.1	98.4
*h*^2^ (%)	Overall	78.0	75.1	46.1	82.4	43.4	58.2	45.3	33.5	91.3
										

1 1 = round to 5 = long;

2 1 = deep to 5 = shallow;

3 1 = none to 5 = heavy; and

4 1 = poor, 5 = excellent; L/W = length–width ratio.

Significant variation was found between market groups for various tuber traits (Table 2). For example, the tuber shape score was significantly higher in a russet market group (4.0) than in chips and reds (1.9 and 1.7, respectively). The degree of russeting was significantly higher in russets than in chips, yellows, purples, and reds. The russets also had a significantly lower average tuber number per plant and higher average weight per tuber than other market groups (Table 2).

**Table 2:** Mean BLUE values of 214 potatoes (by market group) for tuber morphological traits evaluated in three environments in Texas: Dalhart 2019, 2020, and Springlake 2020.

Market group (no of clones)	Tuber shape^1^	L/W	Eye depth^2^	Degree of russeting^3^	Grading score^4^	Av. weight per tuber	Av. tubers per plant	Av. tuber weight per plant	Flesh color
Russets (31)	4.0^a^	1.8^a^	3.9^b^	3.9^a^	3.7^a^	147.8^a^	5.5^c^	790.9^b^	13.2^e^
Chips (62)	1.9^c^	1.2^c^	4.0^ab^	1.3^b^	3.6^b^	121.1^b^	7.9^b^	889.0^a^	15.3^d^
Yellows (32)	1.8^d^	1.2^c^	4.0^ab^	1.0^c^	3.4^c^	76.5^c^	10.1^a^	722.3^bc^	27.2^a^
Purples (21)	2.5^b^	1.5^b^	4.1^a^	1.0^c^	3.5^bc^	76.9^c^	9.2^a^	655.8^c^	21.0^b^
Reds (68)	1.7^d^	1.1^d^	4.0^b^	1.0^c^	3.5^b^	82.3^c^	9.9^a^	788.0^b^	19.6^c^

Values followed by the same letter (within each column) were not significantly different based on Tukey’s tests (*p* < 0.05);

1 1 = round to 5 = long;

2 1 = deep to 5 = shallow;

3 1 = none to 5 = heavy; and

4 1 = poor, 5 = excellent; L/W = length–width ratio.

Analysis of variance showed that clones were significantly different in tuber morphological traits (Table 3). Likewise, the interactions were significant between clone and environments, clone and location, and clone and year for tuber traits (Table 3). Analysis of variance with the data from two sites (DAL 2020 and SPR 2020) indicated that there was a significant difference between locations for tuber shape, length–width ratio, degree of russeting, eye depth, the average weight per tuber, average tuber weight per plant, and grading score (Table 3). For example, the average tuber weight was significantly higher in Dalhart (125.6 g) than Springlake (88.1 g). However average tubers per plant and flesh color were not significantly different when comparing the two locations (Table 3). Analysis of variance with the data from 2 years (DAL 2019 and DAL 2020) indicated that there was no significant difference between years for tuber traits (Table 3). High broad-sense heritability (H > 0.95) was observed for tuber shape, L/W, degree of russeting, and flesh color across three environments (DAL 2019, DAL 2020, and SPR 2020; Table 1). The highest narrow-sense heritability (*h*^2^) of 0.91 was for flesh color and the lowest (0.33) was for average tuber weight per plant across three environments (DAL 2019, DAL 2020, and SPR 2020; Table 1).

**Table 3:** Analysis of variance assessing the effect of **(A)** environments (Env), **(B)** locations (Loc), and **(C)** years on the tuber morphology traits in 214 tetraploid advanced potato clones evaluated in Texas.

Source of variation	Tuber shape	L/W	Eye depth	Degree of russeting	Grading score	Av. weight per tuber	Av. tubers per plant	Av. tuber weight per plant	Flesh color
A. Environments (Dalhart 2019, 2020 and Springlake 2020)									
Clone	***	***	***	***	***	***	***	***	***
Env	ns	ns	ns	ns	ns	ns	ns	ns	ns
Rep (Env)	ns	***	ns	ns	ns	ns	ns	ns	ns
Clone x Env	***	*	***	***	***	***	***	***	**
B. Locations (Dalhart 2020, Springlake 2020)									
Clone	***	***	***	***	***	***	***	***	***
Loc	**	**	*	*	**	**	ns	**	ns
Rep (Loc)	ns	***	ns	ns	ns	ns	ns	ns	ns
Clone x Loc	***	ns	***	***	***	***	*	***	***
C. Years (Dalhart 2019, Dalhart 2020)									
Clone	***	***	***	***	***	***	***	***	***
Year	ns	ns	ns	ns	ns	ns	ns	ns	ns
Rep (Year)	ns	ns	ns	ns	ns	ns	ns	ns	ns
Clone x Year	***	**	***	***	***	***	**	***	ns

ns - not significant;

* *p < 0.05;*

** *p < 0.01; and*

*** *p < 0.001; L/W = length–width ratio.*

**Figure 2:**
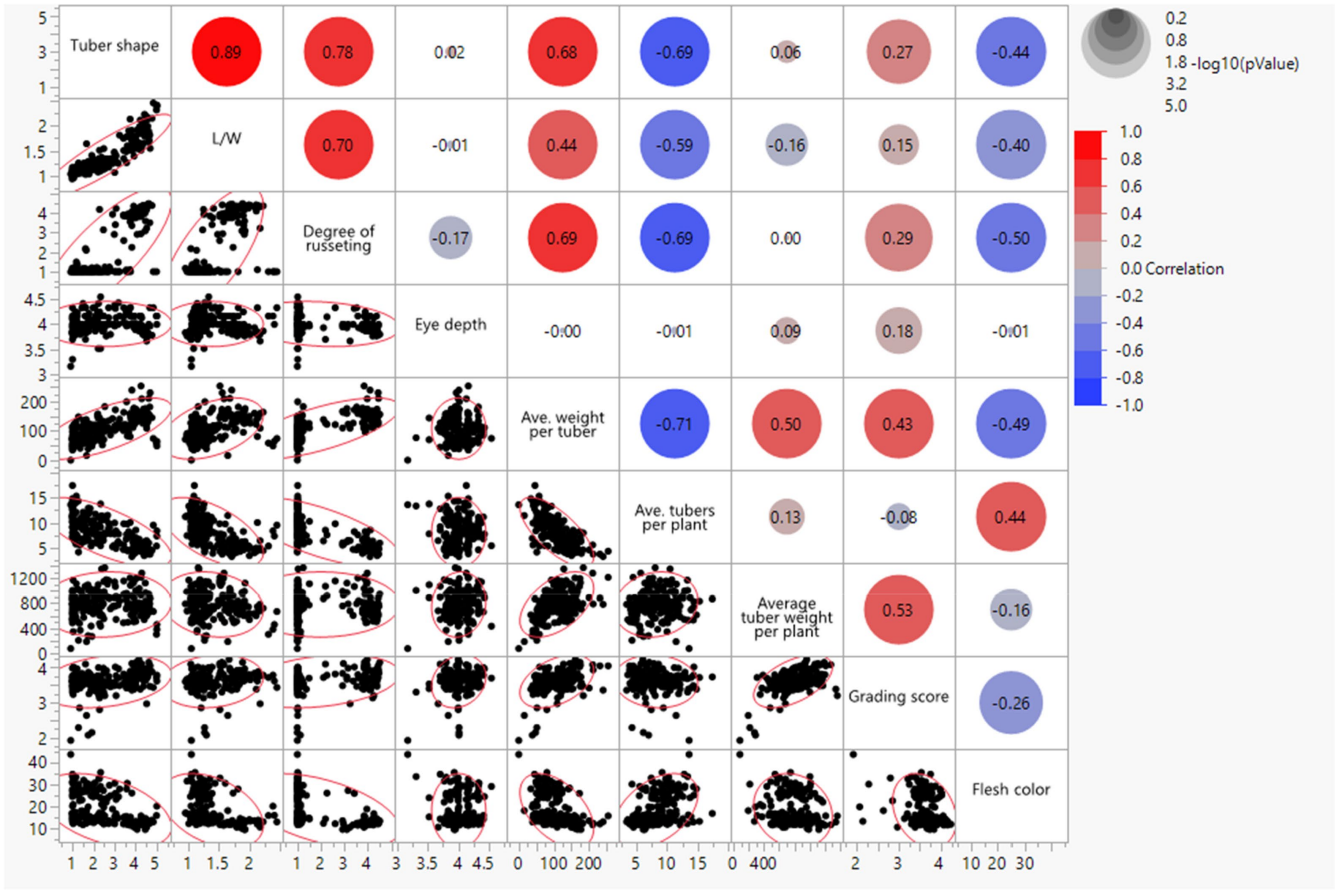
Bivariate scatter plots with a fitted line (bottom of the diagonal) and the value of the correlation (top of the diagonal) for tuber traits of 214 potato clones evaluated in three environments in Texas: Dalhart 2019, 2020, and Springlake 2020.

Pearson correlations (r) for the combined environments were positive between visual tuber shape and L/W ratio (*r* = 0.89; *p* < 0.001), visual tuber shape and russeting (*r* = 0.78; *p* < 0.001; russet potatoes tended to be oblong to long), L/W and russeting (*r* = 0.70; *p* < 0.001; similar interpretation but using L/W objective values), visual tuber shape and average weight per tuber (*r* = 0.68; *p* < 0.001), visual tuber shape and grading score (*r* = 0.27; *p* < 0.001; Figure 2). Negative correlation were found between tuber shape and average tubers per plant (*r* = −0.69; *p* < 0.001; plants with more tubers tended to be rounder), L/W and average tubers per plant (*r* = −0.59; *p* < 0.001), tuber shape and flesh color (*r* = −0.44; *p* < 0.001), L/W and flesh color (*r* = −0.40; *p* < 0.001), average tuber per plant and average weight per tuber (−0.71), average weight per tuber and flesh color (−0.49), grading score and flesh color (−0.26).

### Linkage disequilibrium and GWAS analysis

**Figure 3:**
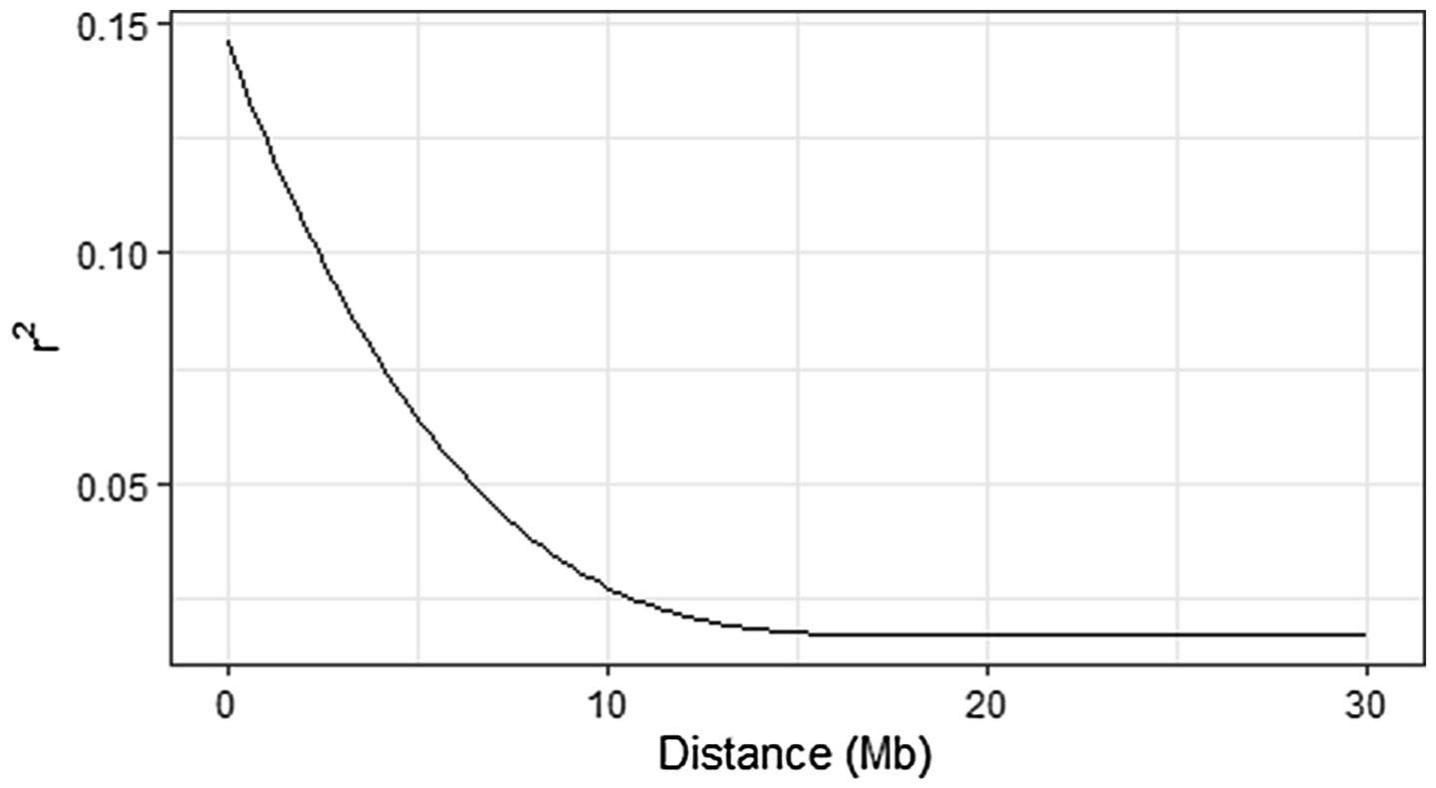
Genome-wide linkage disequilibrium (LD) decay (in Mb) in 214 potato clones using LD. plot function in GWASpoly. A monotone decreasing, convex spline was fit using the R package scam.

**Figure 4:**
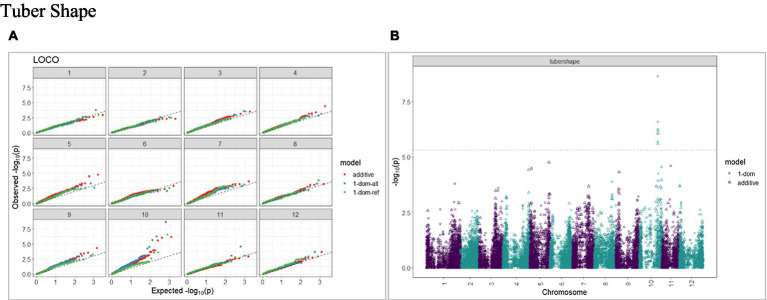
Q–Q plots of observed vs. expected −log10 (*p* values) (**A**) and corresponding Manhattan plot **(B)** for tuber shape using additive and dominant models. GWAS was based on 214 potato clones evaluated for tuber traits of 214 potato clones evaluated in three environments in Texas: Dalhart 2019, 2020, and Springlake 2020. The Bonferroni threshold was at 5.3 for the additive, 5.0 for 1-dom-alt, and 5.1 for the 1-dom-ref model.

**Figure 5:**
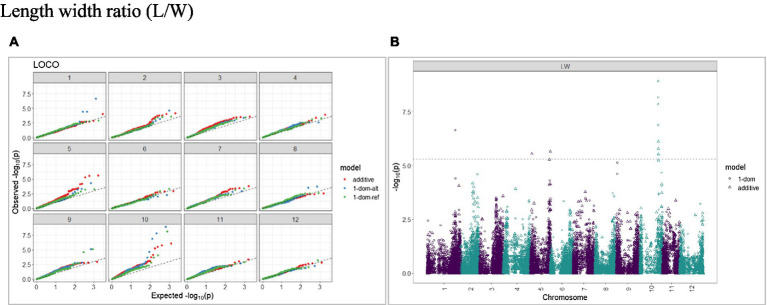
Q–Q plots of observed vs. expected -log10 (*p* values) (**A**) and corresponding Manhattan plot **(B)** for length–width ratio (L/W) using the additive and dominant model for tuber traits of 214 potato clones evaluated in three environments in Texas: Dalhart 2019, 2020 and Springlake 2020. The Bonferroni threshold is at 5.3 for the additive, 5.0 for 1-dom-alt, and 5.1 for the 1-dom-ref model.

**Figure 6:**
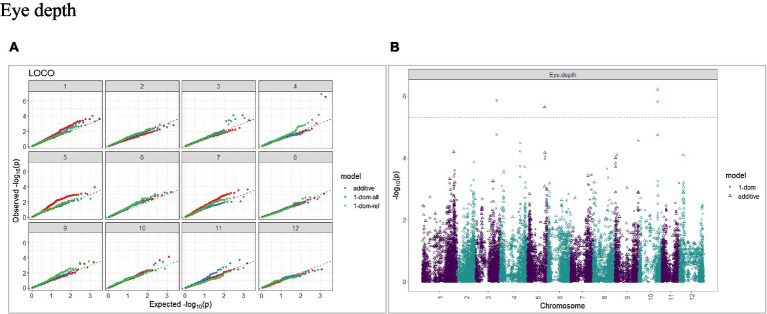
Q–Q plots of observed vs. expected -log10 (*p* values) (**A**) and corresponding Manhattan plot **(B)** for tuber eye depth using the additive and dominant model for tuber traits of 214 potato clones evaluated in three environments in Texas: Dalhart 2019, 2020 and Springlake 2020. The Bonferroni threshold was at 5.3 for the additive, 5.0 for 1-dom-alt, and 5.1 for the 1-dom-ref model.

**Figure 7:**
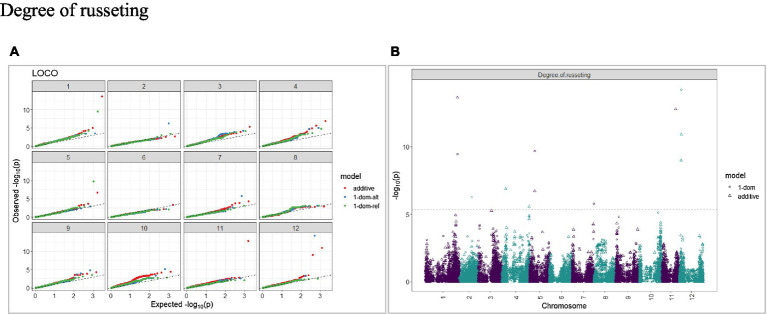
Q–Q plots of observed vs. expected -log10 (*p* values) (**A**) and corresponding Manhattan plot **(B)** for a degree of russeting using the additive and dominant model for tuber traits of 214 potato clones evaluated in three environments in Texas: Dalhart 2019, 2020 and Springlake 2020. The Bonferroni threshold was at 5.3 for the additive, 5.0 for 1-dom-alt, and 5.1 for the 1-dom-ref model.

**Figure 8:**
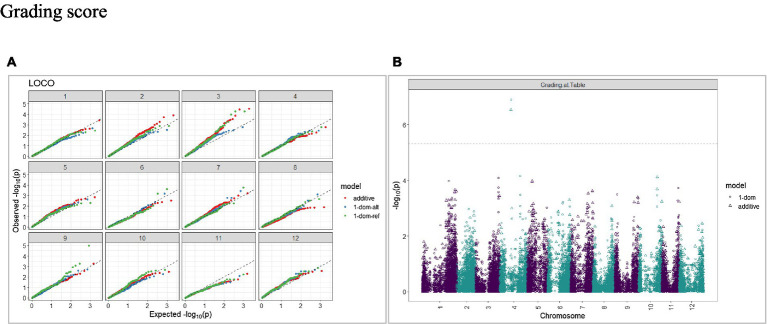
Q–Q plots of observed vs. expected -log10 (*p* values) (**A**) and corresponding Manhattan plot **(B)** for grading score using the additive and dominant model in three combined environments for tuber traits of 214 potato clones evaluated in three environments in Texas: Dalhart 2019, 2020 and Springlake 2020. The Bonferroni threshold was at 5.3 for the additive, 5.0 for 1-dom-alt, and 5.1 for the 1-dom-ref model.

**Figure 9:**
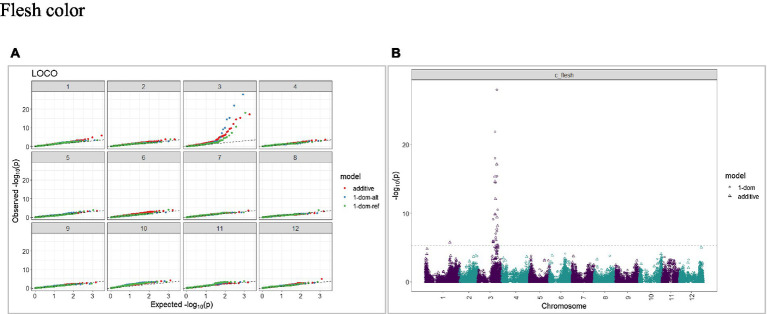
Q–Q plots of observed vs. expected -log10 (*p* values) (**A**) and corresponding Manhattan plot **(B)** for intensity (chroma value) of flesh color using the additive and dominant model for tuber traits of 214 potato clones evaluated in three environments in Texas: Dalhart 2019, 2020 and Springlake 2020. The Bonferroni threshold was at 5.3 for the additive, 5.0 for 1-dom-alt, and 5.1 for the 1-dom-ref model.

**Figure 10:**
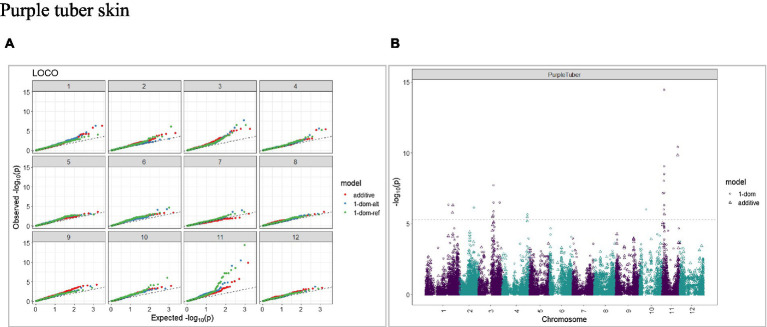
Q–Q plots of observed vs. expected -log10 (*p* values) (**A**) and corresponding Manhattan plot **(B)** for purple skin color using the additive and dominant model for tuber traits of 214 potato clones evaluated in three environments in Texas: Dalhart 2019, 2020 and Springlake 2020. The Bonferroni threshold was at 5.3 for the additive, 5.0 for 1-dom-alt, and 5.1 for the 1-dom-ref model.

The calculated genome-wide LD was low (Figure 3). From the shape of the curve, a 5–10 Mb window seemed appropriate to filter the most significant markers in the output. The inflation of the -log10(p) was examined using a quantile-quantile plot (Q–Q plots) of the observed vs. expected values under the null hypothesis. Significant QTLs were detected for tuber shape (Figure 4), L/W (Figure 5), eye depth (Figure 6), degree of russeting (Figure 7), grading score (Figure 8), the intensity of flesh color (Figure 9) and purple skin color (Figure 10). The marker-trait associations identified in this study were then compared to QTLs reported in previous QTL mapping/GWAS publications (Supplementary Table S1). A QTL on chromosome 10 was identified for tuber shape (based on 1–5 scale) in the additive and the dominant models (1-dom-alt and 1-dom-ref). The SNP at the peak of the QTL was solcap_snp_c2_25485 at a position of 48.7 Mb, explaining 5.8 and 4.0% of the tuber shape variation in the dominant model (1-dom-alt) and additive model, respectively. The SNP solcap_snp_c2_25522 at a position of 48.6 Mb explained 4.3% of the tuber shape variation in the dominant model (1-dom-ref). The SNPs at the peak of the QTLs for tuber shape were near to the genome super scaffold PGSC0003DMB000000385 which annotates as a ribosomal protein S6 kinase. QTLs for L/W coincided with those detected for tuber shape (on chromosome 10), but additional QTLs were identified on chromosomes 1, 5, and 9, explaining between 3.2 to 6.1% of the phenotypic variance. QTLs for eye depth were found on chromosomes 3, 5, and 10. QTLs for a degree of skin russeting were found on all chromosomes, except 3, 6, and 8, and the phenotypic variance explained ranged between 3.2 to 10.5%. A QTL for grading score was identified on chromosome 4 and explained 4.2 and 4.4% of the variation in the additive and dominant model (1-dom-ref), respectively. QTLs for the intensity of flesh color were detected on chromosomes 1 and 3. The SNP PotVar0120627 at a position of 48.6 Mb at the peak of the QTL explained 26.2% of the flesh color variation in the dominant model (1-dom-alt). This SNP marker is near the super scaffold PGSC0003DMG400010169 where the beta-carotene hydroxylase gene (*Bch* or *Chy2*) is located (yellow locus - *Y*). QTLs for purple skin color were detected on chromosomes 1, 2, 3, 4, and 11; and phenotypic variance explained ranged between 9.1 to 25.1%.

### Breeding values of tuber traits

In a breeding program, genomic estimated breeding values (GEBVs) can guide parental selection and the advancement of superior clones. Based on the GEBVs (Supplementary Table S2), clones PTTX05PG07-1 W, COTX08365F-3P/P, and ATX84378-6Ru had among the highest values for long tuber shape, whereas clones COTX10138-15Wpe/Y, NDTX5003-2R, and ATX91322-2Y/Y were among the clones with the lowest GEBVs for tuber shape (best for rounder tuber shape). Clone TX08385-2P/YP had the lowest GEBV for a degree of russeting (best for smooth skin) and clone AOTX95265-4Ru had the highest GEBV for a degree of russeting. Clone ATX91322-2Y/Y had the highest GEBV for eye depth (shallow eyes). ATX84706-2Ru, NDTX092238Cs-1P/W, and ATTX95490-2 W were among the clones with the highest GEBVs for average tuber weight, average tubers per plant, and average tuber weight per plant, respectively. Clone TX13590-9Ru had the highest GEBV for grading score. For practical applications, GEBVs should be considered in the context of market group and selection criteria. Using multi-trait standardized (Z scores) and weighted (depending on the importance of each trait for the market group) GEBVs should contribute to improving selection efficiency and save time and resources required for tuber multiplication and phenotyping.

## Discussion

Breeding for tuber traits in potato can be done more efficiently if there is knowledge of the heritability and genetic basis of tuber morphological characteristics. Molecular markers together with phenotypic evaluations provide efficient and economical mean not only to detect marker-trait associations, but also to obtain GEBVs and to select (early in the breeding process) potato clones carrying desirable traits (as parents and/or generation advancement).

In this study, we identified QTLs associated with tuber shape, eye depth, degree of russeting, tuber number, tuber weight, skin color, and flesh color. There was significant variation for most evaluated traits. The phenotypic distribution was not normal for several tuber traits. A common approach to analyzing non-normally distributed traits is to transform the data (Feng et al., 2014). An alternative to data transformation, often in agricultural research, is to analyze the datasets using mixed models (Piepho et al., 2003).

Strong correlations existed between several of the tuber morphological traits evaluated. Prashar et al. (2014) used a 1–4 ‘breeder’s scale’ and a more ‘quantitative LW’ method to evaluate tuber shape and achieved a correlation coefficient of 0.91 between the two scoring methods. Our study found a correlation of 0.89 between ‘assessed’ (1–5) tuber shape and ‘measured’ (L/W) ratio. The same QTL (on chromosome 10) was identified for ‘assessed’ and ‘measured’ tuber shape traits through GWAS (Supplementary Table S1). The fact that high correlation was obtained between the two traits used to evaluate tuber shape and that the same QTL was identified implies that visual tuber shape evaluation based on a 1–5 scale - despite being less quantitative and more subjective- was appropriate to evaluate tuber shape fast and practically in the potato breeding program. The L/W ratio used in this study was more quantitative and objective (barcode ruler and automated data transfer to a computer), but it was limited to measuring five tubers per replication from one grading class (113.4–170.1 g grading group) and could not represent the whole plot (smaller tubers within a plot tend to be rounder than large tubers). More precise L/W ratios could be obtained if all tubers in the plot are evaluated and high-throughput analysis is done *via* digital image-based methods (Si et al., 2017; Przybylak et al., 2020). Depending on the interest, capacity, and goal of the breeding program, automated digital imaging and appropriate software should be considered to evaluate tuber shape based on L/W but also other traits like skin color, skin texture, eye depth, eye number, and skin damage. Most of the russet potatoes are oblong to long (Table 2), so the correlation coefficient of russeting was high with tuber shape and L/W. A negative correlation (*r* = −0.69) was observed between tuber shape and tubers per plant which means that if tubers were round, there were more tubers per plant, and if tubers were elongated, there were fewer tubers per plant. Understanding tuber characteristics will make visual and indirect selection (based on molecular markers alone) in potato breeding programs more efficient.

High broad-sense heritability was observed for tuber shape, L/W, degree of russeting, and flesh color traits. Love et al. (1997) found broad-sense heritability above 0.80 for russeting. Moderate broad-sense heritability was observed for eye depth and average tuber weight per plant. Likewise, narrow-sense heritability also referred to as “SNP-based heritability,” was the highest (*h*^2^ = 0.82) for russeting compared to that of other traits. The lower heritability estimates can be due to the underrepresentation of significant causal loci by markers (Speed et al., 2012) or dominance. Sood et al. (2020) showed that narrow-sense heritability was moderate for eye depth, average tuber weight, and average tubers per plant. In our study, we obtained similar results. High narrow-sense heritability means that most of the phenotypic variation is genetically controlled by additive effects, and that selection should be successful (Ozturk and Yildirim, 2014; Ozimati et al., 2019). Hence, selecting traits with high narrow-sense heritability would be extremely useful in a breeding program aimed at exploiting continuous genetic gain.

A panel of 214 tetraploid potato clones was genotyped with a high-density SNP marker array and phenotyped for tuber morphological traits at three environments in Texas. A GWAS approach optimized for polyploid species (GWASpoly) was implemented to find marker-trait associations. Some of the QTLs detected for tuber morphological traits in this study coincided with those identified in previous studies, whereas others were discovered for the first time. We report a significant QTL on chromosome 10 (SNP at peak: solcap_snp_c2_25485 at a position of 48.7 Mb) associated with tuber shape and explaining 5.8% of the phenotypic variance in the dominant model. Zia et al. (2020) also detected the same QTL (SNP at peak: solcap_snp_c2_25485) associated with tuber shape. Previous bi-parental mapping studies (Prashar et al., 2014; Hara-Skrzypiec et al., 2018) mapped a major QTL for tuber shape to the same location on chromosome 10. The *Ro* locus on chromosome 10 has been identified as the major locus controlling tuber shape (van Eck et al., 1994a). GWAS conducted by Sharma et al. (2018) reported a QTL (SNP at peak: solcap_snp_c1_8019) located at 48.9 Mb on chromosome 10. In addition to the QTLs identified for the tuber shape, we reported additional QTLs on chromosomes 1, 5, and 9 for L/W. Li et al. (2005) reported that the locus *Ro* was closely linked with a major locus for eye depth. In our study, we also found a QTL on chromosome 10 for eye depth that co-localized with tuber shape.

Early studies by Clark (1933) discovered that the action of complementary factors resulted in the russet skin texture in tubers, but the number of genes involved was not indicated. Few subsequent studies attempted to identify the number of QTLs/genes involved in the russeting of potato tuber skins. Multiple QTLs on chromosomes 1, 2, 4, 5, 7, 10, 11, and 12 were identified for russeting in this study.

In our study, a QTL for flesh color (chroma value) was observed on chromosome 3 at 48.5 Mb (SNP at peak: PotVar0120627) and another QTL with peak at 43.9 Mb (PotVar0070260). The most significant QTL (SNPs at peak: PotVar0120627 and solcap_snp_c2_17552) explained 26 and 14% of the trait variance, respectively. In most tetraploid potato cultivars, the flesh color varies from white to dark yellow due to varying amounts of carotenoids which convey the yellow color. The dominant *Y*-locus is largely responsible for the yellow flesh color of potatoes, and it maps to chromosome 3 (Bonierbale et al., 1988; Thorup et al., 2000). Beta-carotene hydroxylase (*Bch or Chy2*) is the most likely candidate gene responsible for the yellow flesh color (yellow locus - *Y*) (Brown et al., 2006; Kloosterman et al., 2010). One isoform (PGSC0003DMG400009501) of the *Bch* gene is located at 44.1 Mb on chromosome 3 (Xu et al., 2011).

The presence of anthocyanins in a limited number of cultivars results in red or blue/purple flesh color (Bonar et al., 2018). The detection of genomic regions and desirable alleles linked to anthocyanin content could be used in potato breeding programs to increase bioactive compounds. In our study, QTLs for purple skin color were detected on various chromosomes, including chromosome 1, peak at 69.8 Mb (additive model) and chromosome 11 at 4.3 Mb and 39.4 Mb (additive model). Parra-Galindo et al. (2019) detected QTLs associated with cyanidin and delphinidin concentrations on chromosome 1 at 50.7 Mb and chromosome 11 at 40 Mb, respectively. Previous studies showed that the potato *P* locus is required to produce blue/purple anthocyanin pigments and it is located on chromosome 11 (van Eck et al., 1993; De Jong et al., 2004; Jung et al., 2005).

No QTLs were detected for yield-related traits like average weight per tuber, average tubers per plant, and average tuber weight per plant. To acquire appropriate statistical power, GWAS requires much larger population sizes (Hong and Park, 2012). This study was based on only 214 potato clones. In addition, yield-related traits have large phenotypic variation and are highly affected by the environments. Thus, GWAS did not uncover the causative loci. Korte and Farlow (2013) indicated that GWAS often has difficulties in detecting more subtle or multiple QTLs.

Plant breeding is a continuous process that involves the selection and recombination of superior lines. The most important traits in potato breeding are complex and controlled by several genes. Slater et al. (2014) demonstrated the advantage of GEBVs for effective selection for tuber traits in potato breeding. To utilize the GEBVs for selection purposes, the traits may be combined into an index of overall merit. It is feasible to distinguish superior and inferior genotypes by integrating multiple traits using a multiple-trait selection index (Bernardo, 2020). The best index to use depends on the market needs and the objectives of the breeding program. Generating Z-indexes (Mendes et al., 2009; standardized/normalized) has been used as an alternative to identify superior lines in common bean (Lima et al., 2015), upland rice (Ribeiro et al., 2016), and quince cultivars (Coutinho et al., 2019). The incorporation of genomic selection into applied breeding programs in potato appears to be very promising. However, the combination of traits in a multi-trait index and assigning relative weight to each trait still bring some subjectivity on the part of the breeders (relative importance of traits considering the market demands for each class, industry needs, environmental factors and disease/pest considerations) and would benefit from developing models that consider different scenarios and guide the selection process to maximize selection efficiencies and genetic gains.

## Conclusion

The main purpose of the current study was to find QTLs associated with tuber shape, eye depth, degree of russeting, tuber number, tuber weight, skin color, and flesh color in potatoes. Besides the rediscovery of QTL for tuber morphology traits in potatoes, we identified new loci affecting the variation for the studied traits. The genomic estimated breeding values for clones could be used to develop a standardized and weighted selection index for multiple traits which can then be applied to each market group separately.

## Data availability statement

The raw data supporting the conclusions of this article will be made available by the authors, without undue reservation.

## Author contributions

JP: conceptualization, original draft, data collection and analysis, and review and editing. DS and JK: data collection and review and editing. MV: conceptualization, funding acquisition, project administration, supervision, data collection and analysis, and review and editing. All authors contributed to the article and approved the submitted version.

## Funding

This research work was supported by funding from USDA-NIFA (grant no: 2019-34141-35449) and USDA-NIFA-SCRI (award no: 2020-51181-32156).

## Conflict of interest

The authors declare that the research was conducted in the absence of any commercial or financial relationships that could be construed as a potential conflict of interest.

## Publisher’s note

All claims expressed in this article are solely those of the authors and do not necessarily represent those of their affiliated organizations, or those of the publisher, the editors and the reviewers. Any product that may be evaluated in this article, or claim that may be made by its manufacturer, is not guaranteed or endorsed by the publisher.

## Footnotes

^1^Pandey, J., Scheuring, D. C., Koym, J. W., Endelman, J.B., and Vales, M. I. (2022). Genomic selection and genome-wide association studies in tetraploid chipping potatoes. Unpublished manuscript.
