# Corrigendum: Genomic regions associated with tuber traits in tetraploid potatoes and identification of superior clones for breeding purposes

**DOI:** 10.3389/fpls.2024.1396479

**Published:** 2024-03-22

**Authors:** Jeewan Pandey, Douglas C. Scheuring, Jeffrey W. Koym, M. Isabel Vales

**Affiliations:** ^1^ Department of Horticultural Sciences, Texas A&M University, College Station, TX, United States; ^2^ Texas A&M University AgriLife Research and Extension Center, Lubbock, TX, United States

**Keywords:** tuber morphology, genome-wide association studies, single-nucleotide polymorphism, *Solanum tuberosum* ssp. *tuberosum* L., genomic prediction

**Text Correction**

In the published article, there was an error. Due to a misreading of the QTL position for eye depth, we mentioned that a QTL for eye depth was identified near the *CDF1* gene.

A correction has been made to the Abstract, Discussion, and Reference sections.

The following sentences have been removed from the abstract and discussion:

Abstract: “Another QTL peak for eye depth on chromosome 5 was located near the *CDF1* gene, an important regulator of maturity in potato”.

Discussion (Paragraph number 5): “The SNP at the peak of the QTL for eye depth on chromosome 5 is located near the *CDF1* gene, an important regulator of maturity in potato (Kloosterman et al., 2013)”.

The following reference has been removed:

Kloosterman, B., Abelenda, J. A., Gomez, M. D. M. C., Oortwijn, M., de Boer, J. M., and Kowitwanich, K. (2013). Naturally occurring allele diversity allows potato cultivation in northern latitudes. *Nature* 495, 246–250. doi: 10.1038/nature11912

The authors apologize for this error and state that this does not change the scientific conclusions of the article in any way. The original article has been updated.

