# Supplementary material for: Genomic regions associated with tuber traits in tetraploid potatoes and identification of superior clones for breeding purposes
Source: Front Plant Sci. 2022 Jul 22;13:952263. doi: 10.3389/fpls.2022.952263 (PMC9354404; doi:10.3389/fpls.2022.952263)
Supplement: Supplementary file 2 [file Table_2.docx]

Table S2. Genomic-estimated breeding values (GEBVs) of 214 advanced tetraploid potato clones for tuber morphology traits.

| Clones* | Tuber  shape^1^ | L/W | Eye  depth^2^ | Degree of  russeting^3^ | Grading  score^4^ | Av. weight  per tuber | Av. tubers  per plant | Av. tuber  weight per plant | Flesh  color |
| --- | --- | --- | --- | --- | --- | --- | --- | --- | --- |
|  | (1-5) | (ratio) | (1-5) | (1-5) | (1-5) | (g) | (no) | (g) | (chroma value) |
| AOR07781-2 | 3.7 | 1.6 | 4.1 | 3.4 | 3.8 | 146.9 | 7.4 | 1029.9 | 11.7 |
| AORTX09037-1W/Y | 1.5 | 1.2 | 4.2 | 1.0 | 3.7 | 113.9 | 9.1 | 870.8 | 23.0 |
| AOTX02136-1Ru | 3.9 | 1.7 | 4.2 | 3.8 | 3.7 | 152.0 | 5.6 | 821.7 | 12.5 |
| AOTX03187-1Ru | 4.1 | 2.2 | 4.3 | 1.3 | 3.5 | 83.7 | 8.9 | 717.1 | 12.4 |
| AOTX05043-1Ru | 3.6 | 1.6 | 4.0 | 3.9 | 3.6 | 119.6 | 5.2 | 633.4 | 11.6 |
| AOTX91861-4R | 1.1 | 1.1 | 3.8 | 1.1 | 3.7 | 107.2 | 8.9 | 927.6 | 14.4 |
| AOTX93483-1R | 2.6 | 1.2 | 4.1 | 1.1 | 3.6 | 100.7 | 9.2 | 812.4 | 13.7 |
| AOTX95265-2Ru | 4.4 | 1.9 | 3.9 | 4.2 | 3.7 | 145.5 | 5.1 | 722.2 | 13.9 |
| AOTX95295-3Ru | 4.5 | 2.0 | 3.8 | 4.3 | 3.8 | 147.1 | 4.9 | 728.8 | 13.4 |
| AOTX95309-1W | 3.3 | 1.3 | 4.0 | 2.3 | 3.5 | 151.6 | 6.4 | 839.5 | 14.9 |
| AOTX95309-2W* | 2.7 | 1.3 | 4.2 | 1.0 | 3.7 | 106.0 | 9.2 | 907.3 | 23.9 |
| AOTX96075-1Ru | 4.4 | 2.0 | 3.8 | 4.3 | 3.8 | 147.0 | 4.9 | 727.8 | 13.3 |
| AOTX96084-1Ru | 4.4 | 1.9 | 3.8 | 4.2 | 3.7 | 140.4 | 5.2 | 717.2 | 13.5 |
| AOTX96208-1Ru | 4.3 | 1.9 | 3.8 | 4.2 | 3.8 | 143.2 | 5.1 | 722.6 | 13.0 |
| AOTX96216-1Ru | 4.4 | 1.6 | 3.9 | 4.3 | 3.8 | 191.6 | 4.4 | 815.8 | 12.5 |
| AOTX96216-2Ru | 4.5 | 1.6 | 4.0 | 4.3 | 3.8 | 194.8 | 4.4 | 813.9 | 12.6 |
| AOTX97213-1Ru | 4.0 | 1.8 | 4.0 | 3.8 | 3.7 | 132.0 | 6.5 | 839.7 | 13.0 |
| AOTX98096-1Ru* | 4.4 | 2.0 | 3.8 | 4.3 | 3.8 | 146.7 | 4.9 | 729.0 | 13.3 |
| AOTX98137-1Ru | 4.5 | 2.0 | 3.8 | 4.3 | 3.8 | 146.9 | 4.9 | 728.1 | 13.3 |
| AOTX98152-3Ru | 4.0 | 1.5 | 4.0 | 3.8 | 4.0 | 169.7 | 6.7 | 1109.3 | 11.6 |
| AOTX98202-1Ru | 4.1 | 1.7 | 4.1 | 3.7 | 3.8 | 142.1 | 6.7 | 879.5 | 12.8 |
| Atlantic | 1.7 | 1.1 | 3.8 | 2.1 | 3.8 | 125.3 | 7.8 | 968.0 | 14.6 |
| ATTX00289-4W | 2.1 | 1.2 | 4.1 | 1.1 | 3.7 | 124.1 | 8.0 | 956.6 | 17.0 |
| ATTX00289-5R/Y | 2.5 | 1.2 | 3.9 | 1.0 | 3.5 | 108.4 | 10.5 | 1060.3 | 22.6 |
| ATTX00289-6Y/Y | 2.0 | 1.2 | 4.2 | 1.1 | 3.5 | 112.7 | 8.4 | 887.6 | 30.8 |
| ATTX01178-1R | 1.1 | 1.1 | 4.0 | 1.0 | 3.7 | 106.5 | 9.0 | 882.9 | 14.6 |
| ATTX01180-1R/Y | 2.6 | 1.3 | 4.0 | 1.1 | 3.5 | 91.2 | 10.1 | 828.0 | 28.9 |
| ATTX03516-2R | 1.5 | 1.1 | 4.0 | 1.0 | 3.7 | 98.6 | 8.6 | 823.2 | 18.0 |
| ATTX05175s-1R/Y | 1.0 | 1.0 | 3.8 | 1.0 | 3.6 | 48.1 | 12.0 | 588.5 | 34.4 |
| ATTX05186-2R | 1.1 | 1.1 | 4.0 | 1.0 | 3.6 | 54.8 | 12.3 | 674.4 | 14.3 |
| ATTX06246-1R | 1.3 | 1.2 | 4.1 | 1.1 | 3.6 | 63.1 | 12.2 | 740.5 | 15.3 |
| ATTX07042-3W | 1.3 | 1.1 | 4.0 | 1.4 | 3.4 | 114.2 | 7.7 | 818.7 | 24.7 |
| ATTX10265-4R/Y | 1.7 | 1.2 | 4.1 | 1.0 | 3.6 | 76.0 | 10.4 | 773.9 | 30.4 |
| ATTX88481-1P/W | 3.5 | 1.4 | 4.2 | 1.1 | 3.8 | 128.9 | 7.7 | 927.2 | 15.3 |
| ATTX88654-2P/Y | 1.2 | 1.0 | 3.8 | 1.0 | 3.4 | 85.5 | 8.5 | 674.9 | 31.4 |
| ATTX95490-2W | 2.3 | 1.2 | 3.9 | 1.0 | 3.4 | 120.0 | 9.7 | 1115.4 | 13.6 |
| ATTX961014-1AR/Y | 2.7 | 1.3 | 4.1 | 1.0 | 3.8 | 109.7 | 8.9 | 909.0 | 24.1 |
| ATTX961014-1Br/Y | 2.7 | 1.3 | 4.2 | 1.0 | 3.7 | 106.0 | 9.1 | 903.7 | 23.9 |
| ATTX961014-1R/Y Chimera | 1.4 | 1.2 | 4.0 | 1.0 | 3.6 | 83.7 | 9.8 | 812.6 | 15.8 |
| ATTX96746-1R* | 2.5 | 1.3 | 4.1 | 1.1 | 3.5 | 89.3 | 7.9 | 703.7 | 13.0 |
| ATTX98444s-16R/Y | 1.3 | 1.2 | 4.0 | 1.1 | 3.2 | 54.2 | 9.7 | 554.6 | 22.7 |
| ATTX98448-6R/Y | 2.5 | 1.2 | 3.9 | 1.0 | 3.5 | 108.9 | 10.5 | 1060.7 | 22.5 |
| ATTX98453-11Br | 1.9 | 1.1 | 3.9 | 1.1 | 3.6 | 77.7 | 11.3 | 802.6 | 13.5 |
| ATTX98453-3R | 1.6 | 1.1 | 3.9 | 1.0 | 3.6 | 82.0 | 11.0 | 875.0 | 12.1 |
| ATTX98453-6R | 1.8 | 1.2 | 4.1 | 1.2 | 3.6 | 108.1 | 8.3 | 816.8 | 12.6 |
| ATTX98462s-3R/Y | 2.7 | 1.3 | 4.2 | 1.0 | 3.8 | 106.6 | 9.1 | 908.5 | 24.0 |
| ATTX98465-1R/Y | 1.1 | 1.1 | 3.5 | 1.1 | 3.3 | 80.5 | 12.4 | 923.5 | 33.0 |
| ATTX98466-5R/WR | 1.4 | 1.1 | 4.0 | 1.0 | 3.6 | 83.3 | 9.8 | 812.4 | 14.8 |
| ATTX98468-5Ru/Y* | 3.9 | 1.8 | 4.1 | 4.1 | 3.6 | 133.6 | 4.8 | 615.6 | 12.3 |
| ATTX98491-4Yrdspl/Y | 1.7 | 1.1 | 3.8 | 1.0 | 3.4 | 69.7 | 10.2 | 699.2 | 34.1 |
| ATTX98493-1AR | 2.0 | 1.2 | 4.0 | 1.1 | 3.5 | 77.8 | 10.3 | 776.3 | 26.9 |
| ATTX98493-2P/P | 1.9 | 1.3 | 4.2 | 1.2 | 3.6 | 78.6 | 9.7 | 717.1 | 16.5 |
| ATTX98500-2P/Y | 1.6 | 1.2 | 4.0 | 1.0 | 3.4 | 64.7 | 9.4 | 640.1 | 31.5 |
| ATTX98500-3P/Y | 2.2 | 1.4 | 4.0 | 1.1 | 3.0 | 73.3 | 7.9 | 549.2 | 33.2 |
| ATTX98510-1R/Y | 1.4 | 1.1 | 3.9 | 1.0 | 3.5 | 82.3 | 9.7 | 780.1 | 26.1 |
| ATTX98514-1R/Y | 1.8 | 1.2 | 4.0 | 1.0 | 3.4 | 81.1 | 6.8 | 530.5 | 25.0 |
| ATTX98518-5P/Y | 3.2 | 1.7 | 4.1 | 1.0 | 3.5 | 128.7 | 7.3 | 839.5 | 28.0 |
| ATTX99325-1P | 3.5 | 1.4 | 4.2 | 1.1 | 3.8 | 127.2 | 7.6 | 924.3 | 15.5 |
| ATX02263-1R/Y | 2.0 | 1.3 | 4.2 | 1.0 | 3.7 | 68.0 | 9.6 | 664.8 | 23.8 |
| ATX03496-3Y/Y | 2.3 | 1.3 | 4.1 | 1.1 | 3.5 | 87.3 | 10.0 | 744.4 | 24.7 |
| ATX03564-1Y/Y | 2.3 | 1.3 | 4.1 | 1.0 | 3.5 | 87.1 | 10.0 | 744.4 | 24.2 |
| ATX05186-1R | 1.2 | 1.1 | 4.0 | 0.9 | 3.6 | 54.6 | 12.8 | 690.7 | 14.2 |
| ATX05202s-3W/Y | 1.0 | 1.1 | 4.2 | 1.1 | 3.7 | 67.2 | 11.1 | 740.4 | 26.6 |
| ATX06264s-4R/Y | 1.8 | 1.2 | 4.1 | 1.0 | 3.3 | 65.7 | 11.2 | 699.6 | 29.6 |
| ATX07305S-1Y/Y | 1.2 | 1.1 | 3.9 | 1.1 | 3.4 | 54.2 | 11.6 | 632.3 | 25.7 |
| ATX08181-5Y/Y | 1.0 | 1.1 | 4.1 | 1.1 | 3.5 | 42.5 | 13.3 | 629.9 | 27.8 |
| ATX84378-6Ru | 4.7 | 1.6 | 4.0 | 4.4 | 3.9 | 208.9 | 4.2 | 855.1 | 11.9 |
| ATX84706-2Ru | 4.2 | 1.5 | 4.2 | 3.6 | 4.0 | 226.6 | 4.5 | 1072.7 | 15.7 |
| ATX85404-8W | 3.3 | 1.3 | 4.0 | 2.4 | 3.5 | 152.2 | 6.4 | 840.3 | 14.8 |
| ATX87184-2Ru | 4.0 | 1.5 | 4.0 | 3.7 | 3.6 | 174.1 | 4.1 | 761.2 | 12.8 |
| ATX9117-1Ru | 3.8 | 1.6 | 4.0 | 3.6 | 3.4 | 151.1 | 4.6 | 670.4 | 12.7 |
| ATX9130-1Ru | 4.6 | 1.8 | 4.3 | 3.6 | 3.5 | 173.1 | 5.1 | 846.4 | 12.7 |
| ATX91322-2Y/Y | 1.0 | 1.1 | 3.3 | 1.0 | 2.4 | 10.1 | 13.3 | 246.7 | 44.0 |
| ATX9202-3Ru | 3.5 | 1.8 | 4.1 | 3.8 | 3.6 | 108.7 | 5.7 | 618.8 | 11.7 |
| ATX9312-1Ru | 4.0 | 1.8 | 3.9 | 4.1 | 3.5 | 129.2 | 5.2 | 656.6 | 11.7 |
| ATX9332-8Ru | 4.0 | 1.8 | 3.9 | 3.8 | 3.5 | 145.5 | 4.4 | 670.2 | 10.7 |
| ATX97147-4Ru | 4.0 | 1.7 | 4.0 | 3.9 | 3.6 | 137.4 | 5.4 | 723.5 | 12.1 |
| ATX99013-1Ru | 4.5 | 2.0 | 3.8 | 4.3 | 3.8 | 147.0 | 4.9 | 729.0 | 13.3 |
| BTX1544-2W/Y | 2.2 | 1.3 | 4.0 | 1.3 | 3.5 | 104.5 | 8.0 | 823.0 | 26.2 |
| BTX1749-1W/Y | 1.7 | 1.2 | 4.2 | 1.1 | 3.7 | 83.5 | 10.1 | 843.1 | 29.1 |
| BTX2103-1R/Y | 1.5 | 1.1 | 3.9 | 1.0 | 3.6 | 76.3 | 10.1 | 742.6 | 27.3 |
| BTX2332-1R | 1.2 | 1.1 | 4.2 | 1.0 | 3.8 | 109.0 | 7.9 | 831.9 | 14.7 |
| CO112-F2-2P/P | 1.8 | 1.6 | 3.9 | 1.1 | 3.0 | 35.6 | 10.5 | 392.5 | 18.3 |
| COTX00104-6R | 2.0 | 1.2 | 4.1 | 1.0 | 3.4 | 105.0 | 7.4 | 731.9 | 14.5 |
| COTX00104-7R | 2.4 | 1.2 | 4.0 | 1.0 | 3.5 | 108.2 | 8.2 | 810.1 | 12.9 |
| COTX01403-4R/Y | 3.3 | 1.2 | 4.0 | 1.0 | 3.5 | 100.9 | 8.8 | 860.0 | 27.6 |
| COTX02172-1R | 2.1 | 1.2 | 4.1 | 1.0 | 3.7 | 86.4 | 8.7 | 713.3 | 13.5 |
| COTX02293-4R | 1.4 | 1.2 | 4.0 | 1.0 | 3.7 | 87.9 | 9.4 | 777.3 | 15.1 |
| COTX03079-1W/W | 2.4 | 1.2 | 4.0 | 1.0 | 3.6 | 88.0 | 10.5 | 912.3 | 27.5 |
| COTX03134-1Y/W | 1.9 | 1.3 | 4.0 | 1.1 | 2.8 | 50.8 | 8.5 | 523.6 | 20.5 |
| COTX03187-1W | 4.4 | 2.2 | 4.3 | 1.4 | 3.5 | 84.7 | 8.7 | 715.8 | 12.6 |
| COTX04015-3W/Y | 2.2 | 1.3 | 4.1 | 1.1 | 3.3 | 78.5 | 9.4 | 699.5 | 32.6 |
| COTX04050s-1P/P | 1.2 | 1.1 | 4.1 | 1.1 | 3.6 | 69.5 | 10.3 | 701.4 | 17.2 |
| COTX04193s-2R/Y | 1.2 | 1.1 | 4.1 | 1.0 | 3.6 | 67.7 | 10.5 | 703.4 | 28.8 |
| COTX04303-3Ru/Y* | 4.5 | 2.0 | 3.8 | 4.3 | 3.8 | 146.8 | 4.9 | 728.2 | 13.3 |
| COTX05095-2Ru/Y | 3.8 | 1.8 | 4.1 | 2.4 | 3.7 | 118.8 | 7.3 | 841.0 | 25.6 |
| COTX05211-4R | 2.0 | 1.4 | 4.0 | 1.0 | 3.5 | 72.6 | 9.4 | 687.4 | 14.9 |
| COTX05211-5R | 2.0 | 1.4 | 4.0 | 1.0 | 3.5 | 72.6 | 9.4 | 687.3 | 15.0 |
| COTX05211-7R | 1.2 | 1.1 | 4.0 | 1.0 | 3.5 | 77.1 | 11.1 | 847.8 | 14.1 |
| COTX05249-3W/Y | 1.1 | 1.2 | 4.0 | 1.0 | 3.7 | 61.4 | 11.2 | 679.9 | 25.6 |
| COTX05261-1R/Y | 3.4 | 1.5 | 4.0 | 1.0 | 3.5 | 82.9 | 9.9 | 791.9 | 29.6 |
| COTX08039-1P/P* | 2.4 | 1.5 | 4.2 | 1.0 | 3.4 | 70.3 | 8.9 | 631.8 | 12.3 |
| COTX08063-2Ru | 3.8 | 1.9 | 4.0 | 3.4 | 3.6 | 130.4 | 5.2 | 713.8 | 11.9 |
| COTX08121-1Ru | 3.9 | 1.7 | 4.1 | 3.9 | 3.7 | 145.1 | 5.8 | 812.0 | 12.3 |
| COTX08121-4Ru | 3.5 | 1.7 | 4.0 | 3.8 | 3.7 | 118.6 | 6.3 | 771.8 | 11.7 |
| COTX08258-6Ru | 3.5 | 1.6 | 4.2 | 2.8 | 3.8 | 125.0 | 8.7 | 1009.0 | 12.2 |
| COTX08322-10Ru | 4.1 | 1.6 | 4.0 | 3.9 | 4.0 | 167.8 | 6.4 | 1040.3 | 11.7 |
| COTX08365F-3P/P | 4.7 | 2.1 | 4.0 | 1.1 | 3.6 | 67.2 | 8.6 | 548.7 | 16.0 |
| COTX09022-3RuRE/Y | 2.4 | 1.3 | 4.0 | 3.0 | 3.9 | 118.9 | 7.3 | 836.8 | 26.3 |
| COTX09052-1Ru | 3.7 | 1.7 | 4.1 | 3.7 | 3.7 | 104.6 | 7.4 | 775.7 | 12.5 |
| COTX09052-2Ru | 3.7 | 1.8 | 4.1 | 3.7 | 3.7 | 104.7 | 7.4 | 778.1 | 12.4 |
| COTX09089-1Ru | 2.4 | 1.3 | 4.0 | 3.9 | 3.4 | 114.3 | 6.6 | 782.8 | 14.6 |
| COTX10012-1Wrdspl/Y | 1.5 | 1.2 | 4.0 | 1.1 | 3.7 | 91.9 | 10.0 | 889.0 | 27.6 |
| COTX10073s-1W | 1.2 | 1.2 | 4.1 | 1.1 | 3.5 | 65.7 | 11.1 | 686.8 | 16.2 |
| COTX10080-2Ru | 3.8 | 1.7 | 4.0 | 4.0 | 3.8 | 119.9 | 6.4 | 732.9 | 13.1 |
| COTX10118-1Wre/Y | 1.1 | 1.1 | 3.9 | 1.0 | 3.6 | 70.1 | 11.5 | 781.3 | 25.0 |
| COTX10118-4Wpe/Y | 1.8 | 1.2 | 4.0 | 1.0 | 3.7 | 72.7 | 12.1 | 844.7 | 29.0 |
| COTX10138-15Wpe/Y | 1.0 | 1.1 | 4.1 | 0.9 | 3.8 | 66.6 | 11.9 | 765.2 | 28.1 |
| COTX10138-19P/Y | 1.9 | 1.1 | 4.0 | 1.0 | 3.5 | 111.8 | 8.7 | 814.9 | 28.1 |
| COTX10138s-7Wpe/Y | 1.3 | 1.1 | 4.0 | 0.9 | 3.7 | 65.9 | 12.7 | 814.3 | 28.9 |
| COTX10226-1Wpe/Y | 1.0 | 1.0 | 3.9 | 1.0 | 3.6 | 50.1 | 10.6 | 592.8 | 31.1 |
| COTX13215-2Ru | 3.0 | 1.5 | 4.1 | 2.9 | 3.6 | 115.0 | 6.0 | 792.2 | 13.8 |
| COTX87601-2Ru | 4.4 | 1.8 | 3.9 | 3.9 | 3.9 | 157.7 | 5.4 | 857.1 | 11.9 |
| COTX89044-1Ru | 3.7 | 1.8 | 4.0 | 3.6 | 3.5 | 137.5 | 5.2 | 709.2 | 12.4 |
| COTX90046-1W | 2.2 | 1.2 | 4.0 | 1.2 | 3.7 | 108.6 | 7.4 | 816.7 | 13.6 |
| COTX90046-5W | 3.1 | 1.2 | 4.0 | 1.7 | 3.6 | 128.6 | 6.5 | 832.9 | 14.6 |
| COTX94216-1R | 1.1 | 1.1 | 4.1 | 1.0 | 3.7 | 75.2 | 11.2 | 793.2 | 15.3 |
| COTX94218-1R | 1.0 | 1.1 | 4.0 | 0.9 | 3.6 | 70.4 | 10.4 | 736.2 | 14.7 |
| JTTX75/2003EH-1Yre/Y | 2.9 | 1.4 | 3.9 | 1.5 | 3.5 | 93.1 | 7.8 | 703.3 | 24.7 |
| Krantz | 3.3 | 1.3 | 4.1 | 3.7 | 3.7 | 164.2 | 6.3 | 945.0 | 15.5 |
| MWTX2609-2Ru | 4.3 | 1.9 | 4.0 | 3.0 | 3.7 | 164.2 | 6.3 | 993.5 | 9.7 |
| MWTX2609-4Ru | 4.3 | 1.9 | 4.0 | 3.0 | 3.7 | 164.6 | 6.3 | 995.9 | 9.6 |
| MWTX548-2Ru | 4.3 | 1.9 | 4.0 | 3.0 | 3.7 | 163.5 | 6.3 | 991.2 | 9.8 |
| NDTX050169-1R | 1.0 | 1.1 | 4.0 | 1.1 | 3.6 | 47.1 | 13.4 | 621.8 | 13.1 |
| NDTX050184s-1R/Y | 1.1 | 1.1 | 4.0 | 1.0 | 3.8 | 63.4 | 13.3 | 767.3 | 24.6 |
| NDTX059759-3R/Y Pinto | 2.2 | 1.3 | 4.3 | 0.9 | 3.6 | 73.5 | 7.3 | 567.0 | 28.7 |
| NDTX059761-1R/R | 2.5 | 1.4 | 4.0 | 1.1 | 3.5 | 58.5 | 10.6 | 616.7 | 16.3 |
| NDTX059775-1W | 2.1 | 1.3 | 3.9 | 1.0 | 3.6 | 68.5 | 11.5 | 748.9 | 24.8 |
| NDTX059828-2W | 1.1 | 1.1 | 3.9 | 0.9 | 3.7 | 75.1 | 10.1 | 773.4 | 15.1 |
| NDTX059886S-1Y/Y | 1.7 | 1.2 | 4.1 | 1.1 | 3.6 | 88.4 | 9.3 | 766.4 | 25.1 |
| NDTX060700C-1W | 1.3 | 1.1 | 4.2 | 1.2 | 3.6 | 71.1 | 9.1 | 665.7 | 15.6 |
| NDTX071109C-1W | 1.3 | 1.0 | 3.9 | 1.0 | 3.7 | 139.2 | 7.2 | 941.7 | 15.4 |
| NDTX071217CB-1W/Y | 1.6 | 1.1 | 4.0 | 1.0 | 3.6 | 98.2 | 8.1 | 759.9 | 26.5 |
| NDTX071258B-1R | 1.1 | 1.1 | 3.9 | 1.1 | 3.6 | 65.0 | 10.8 | 671.9 | 16.4 |
| NDTX081451CBs-1Y/Y | 1.8 | 1.3 | 4.1 | 1.2 | 3.7 | 85.7 | 11.4 | 889.7 | 29.4 |
| NDTX081618-1P/P | 2.3 | 1.3 | 4.3 | 1.0 | 3.8 | 86.5 | 9.8 | 815.3 | 14.7 |
| NDTX081644-CAB-2W | 1.0 | 1.1 | 4.1 | 1.0 | 3.4 | 55.2 | 9.1 | 563.1 | 20.5 |
| NDTX081648CB-13W | 1.7 | 1.1 | 4.0 | 1.2 | 3.8 | 104.9 | 9.1 | 964.9 | 15.8 |
| NDTX081648CB-1W | 1.5 | 1.1 | 4.0 | 1.1 | 3.7 | 95.2 | 9.1 | 807.5 | 15.7 |
| NDTX081648CB-4W | 1.2 | 1.1 | 4.0 | 1.2 | 3.6 | 119.2 | 7.5 | 874.1 | 14.6 |
| NDTX091886-3P/P | 2.2 | 1.2 | 4.2 | 1.0 | 3.5 | 73.0 | 7.4 | 550.8 | 17.3 |
| NDTX091908AB-2W | 1.6 | 1.2 | 4.1 | 1.0 | 3.7 | 107.0 | 9.2 | 937.0 | 15.1 |
| NDTX092237C-2P/W | 2.4 | 1.4 | 3.7 | 1.0 | 3.4 | 61.6 | 12.1 | 702.0 | 23.3 |
| NDTX092238Cs-1P/W | 1.0 | 1.1 | 4.1 | 1.0 | 3.6 | 49.0 | 15.2 | 764.4 | 16.6 |
| NDTX4271-5R | 1.2 | 1.1 | 4.0 | 0.9 | 3.7 | 103.6 | 8.9 | 854.7 | 13.9 |
| NDTX4756-1R/Y | 1.4 | 1.1 | 3.9 | 0.9 | 3.5 | 82.6 | 9.7 | 781.4 | 26.2 |
| NDTX4784-7R | 1.1 | 1.1 | 4.0 | 1.0 | 3.7 | 106.4 | 8.7 | 860.7 | 13.8 |
| NDTX4828-2R | 1.2 | 1.1 | 4.0 | 1.1 | 3.6 | 77.4 | 9.4 | 713.9 | 13.6 |
| NDTX4930-5W | 3.4 | 1.2 | 4.0 | 1.1 | 3.6 | 179.1 | 5.4 | 872.8 | 13.5 |
| NDTX5003-2R | 1.0 | 1.0 | 3.9 | 1.1 | 3.7 | 81.4 | 10.7 | 853.5 | 16.9 |
| NDTX5067-2R | 1.0 | 1.0 | 3.9 | 1.2 | 3.7 | 82.5 | 10.7 | 857.2 | 16.9 |
| NDTX5438-11R | 2.1 | 1.2 | 4.0 | 1.1 | 3.6 | 97.4 | 9.3 | 842.2 | 15.6 |
| NDTX6773-1W | 1.1 | 1.1 | 3.9 | 0.9 | 3.7 | 115.1 | 7.5 | 840.8 | 15.4 |
| NDTX731-1R | 1.1 | 1.0 | 3.9 | 1.0 | 3.7 | 97.2 | 11.6 | 1007.9 | 12.6 |
| NDTX7590-3R | 2.9 | 1.3 | 4.2 | 1.0 | 3.5 | 104.7 | 10.1 | 1009.2 | 12.3 |
| NDTX8773-4Ru | 3.2 | 1.5 | 4.1 | 4.1 | 3.5 | 123.0 | 5.5 | 667.1 | 16.6 |
| NDTX91068-11R | 2.4 | 1.1 | 4.1 | 1.1 | 3.6 | 126.4 | 9.0 | 1020.1 | 12.5 |
| PORTX03PG25-2R/R | 4.6 | 2.1 | 4.1 | 1.0 | 3.7 | 53.6 | 9.0 | 509.9 | 19.9 |
| PTTX05PG07-1W | 4.8 | 2.2 | 4.1 | 1.0 | 3.7 | 59.2 | 7.0 | 440.4 | 16.0 |
| Reveille Russet | 3.8 | 1.6 | 4.0 | 3.8 | 3.7 | 137.8 | 5.5 | 744.5 | 12.5 |
| Rio Rojo | 1.5 | 1.2 | 3.9 | 1.0 | 3.5 | 55.2 | 7.1 | 583.3 | 15.6 |
| Russet Burbank | 4.4 | 2.0 | 3.8 | 3.2 | 3.2 | 111.6 | 6.3 | 681.5 | 11.2 |
| Russet Norkotah | 4.5 | 2.0 | 3.8 | 4.3 | 3.8 | 146.9 | 4.9 | 728.1 | 13.3 |
| Russet Norkotah 102 | 4.4 | 2.0 | 3.8 | 4.3 | 3.8 | 146.1 | 4.9 | 727.6 | 13.4 |
| Russet Norkotah 112 | 4.4 | 2.0 | 3.8 | 4.3 | 3.8 | 146.2 | 4.9 | 725.8 | 13.4 |
| Russet Norkotah 223 | 4.4 | 2.0 | 3.8 | 4.3 | 3.8 | 146.7 | 4.9 | 727.9 | 13.4 |
| Russet Norkotah 278 | 4.5 | 2.0 | 3.8 | 4.3 | 3.8 | 146.8 | 4.9 | 727.9 | 13.3 |
| Russet Norkotah 296 | 4.5 | 2.0 | 3.8 | 4.3 | 3.8 | 146.6 | 4.9 | 728.8 | 13.3 |
| Sierra GoldTM | 2.9 | 1.3 | 4.2 | 3.1 | 3.8 | 161.7 | 7.7 | 984.1 | 25.4 |
| Sierra RoseTM | 2.7 | 1.3 | 4.2 | 1.0 | 3.8 | 106.4 | 9.1 | 905.5 | 24.1 |
| Stampede Russet | 4.0 | 1.6 | 3.9 | 3.8 | 4.0 | 157.9 | 6.4 | 907.5 | 12.2 |
| Tacna | 1.9 | 1.3 | 4.0 | 1.1 | 2.8 | 65.0 | 7.1 | 534.6 | 14.0 |
| Tokio | 1.1 | 1.2 | 4.0 | 1.1 | 3.5 | 58.9 | 10.5 | 610.1 | 30.7 |
| TX03196-1W | 1.7 | 1.1 | 4.0 | 1.0 | 3.6 | 106.3 | 8.4 | 858.3 | 14.5 |
| TX05249-10W | 1.7 | 1.1 | 4.0 | 2.0 | 3.7 | 137.8 | 6.7 | 882.6 | 12.4 |
| TX05249-11W | 1.6 | 1.2 | 4.3 | 1.4 | 3.8 | 109.9 | 6.1 | 721.1 | 15.8 |
| TX05249-3W | 1.4 | 1.1 | 4.1 | 1.4 | 3.6 | 131.2 | 5.2 | 744.2 | 14.6 |
| TX08350-12Ru | 4.3 | 1.6 | 4.0 | 3.1 | 3.9 | 160.5 | 5.8 | 923.2 | 12.3 |
| TX08385-2P/YP | 2.2 | 1.1 | 4.1 | 0.8 | 3.5 | 78.2 | 8.4 | 657.6 | 23.8 |
| TX09396-1W | 1.5 | 1.1 | 3.9 | 1.3 | 3.5 | 132.3 | 6.5 | 838.2 | 16.1 |
| TX09403-15W | 2.3 | 1.1 | 4.0 | 1.2 | 3.5 | 151.8 | 7.7 | 1066.2 | 15.4 |
| TX09403-21W | 2.3 | 1.1 | 4.0 | 1.2 | 3.5 | 154.0 | 7.7 | 1075.7 | 15.6 |
| TX09414-1W | 2.2 | 1.2 | 4.0 | 1.3 | 3.5 | 124.0 | 6.3 | 773.0 | 14.2 |
| TX10437-9Pyspl/Y | 1.5 | 1.3 | 4.0 | 1.0 | 2.9 | 66.3 | 7.5 | 535.1 | 29.0 |
| TX11454-9Ru/Y | 2.9 | 1.3 | 4.0 | 3.7 | 3.6 | 142.3 | 5.7 | 793.0 | 19.0 |
| TX11461-2W | 1.3 | 1.1 | 4.0 | 1.4 | 3.6 | 112.3 | 9.3 | 988.0 | 14.6 |
| TX11461-3W | 1.9 | 1.1 | 4.0 | 1.2 | 3.6 | 105.1 | 9.4 | 942.4 | 15.0 |
| TX12474-1P/R | 1.1 | 1.2 | 4.1 | 1.1 | 3.4 | 43.5 | 9.8 | 448.2 | 20.6 |
| TX12484-4W | 2.0 | 1.2 | 4.0 | 1.2 | 3.7 | 96.0 | 8.8 | 799.2 | 12.8 |
| TX13590-9Ru | 4.0 | 1.5 | 4.0 | 3.9 | 4.0 | 168.9 | 6.8 | 1106.5 | 11.5 |
| TX14611-1R | 1.0 | 1.1 | 4.1 | 1.0 | 3.6 | 74.7 | 8.6 | 627.9 | 16.3 |
| TX1475-3W | 2.2 | 1.1 | 3.9 | 1.3 | 3.7 | 151.4 | 6.1 | 915.0 | 15.7 |
| TX1617-1W/Y | 3.0 | 1.4 | 4.3 | 1.2 | 3.6 | 112.7 | 7.3 | 835.7 | 30.0 |
| TX1673-1W/Y | 2.3 | 1.2 | 4.1 | 1.1 | 3.7 | 125.1 | 7.9 | 957.9 | 18.3 |
| TX6-1216-1Ru | 4.1 | 1.6 | 3.9 | 3.8 | 3.7 | 151.5 | 5.0 | 761.0 | 11.5 |
| TXA549-1Ru | 4.0 | 1.6 | 4.0 | 3.9 | 4.0 | 169.0 | 6.8 | 1106.1 | 11.5 |
| TXNS106 | 4.4 | 2.0 | 3.8 | 4.3 | 3.8 | 146.9 | 4.9 | 727.5 | 13.3 |
| TXNS118 | 4.5 | 2.0 | 3.8 | 4.3 | 3.8 | 146.7 | 4.9 | 727.5 | 13.3 |
| TXNS249 | 4.4 | 2.0 | 3.8 | 4.3 | 3.8 | 146.3 | 4.9 | 727.1 | 13.3 |
| TXYG79 | 2.2 | 1.2 | 4.1 | 1.2 | 3.6 | 129.2 | 7.2 | 857.7 | 28.1 |
| UMTX383-3Yrdspl/Y | 1.8 | 1.1 | 3.8 | 1.0 | 3.4 | 70.9 | 10.1 | 700.8 | 34.2 |
| Vanguard Russet | 4.0 | 1.7 | 4.0 | 4.1 | 4.0 | 157.7 | 7.0 | 985.4 | 11.7 |
| White LaSoda | 2.3 | 1.2 | 3.8 | 1.2 | 3.6 | 142.6 | 7.8 | 1055.9 | 15.2 |

*^1^1= round to 5= long; ^2^1= deep to 5= shallow; ^3^1= none to 5= heavy; ^4^1= poor, 5= excellent; L/W= length width ratio*

** The clones are maintained in vitro by the Texas A&M Potato Breeding Program. Details about the clones can be found in supplementary tables included in Pandey et al. (2021).*
